# Real-world clinical effectiveness of Tixagevimab/Cilgavimab and Regdanvimab monoclonal antibodies for COVID-19 treatment in Omicron variant-dominant period

**DOI:** 10.3389/fimmu.2023.1259725

**Published:** 2023-10-20

**Authors:** Daria S. Fomina, Marina S. Lebedkina, Anna A. Iliukhina, Anna V. Kovyrshina, Artem Y. Shelkov, Sergey S. Andreev, Anton A. Chernov, Inna V. Dolzhikova, Tatyana S. Kruglova, Gerelma V. Andrenova, Amir I. Tukhvatulin, Dmitry V. Shcheblyakov, Alexander V. Karaulov, Maryana A. Lysenko, Denis Y. Logunov, Alexander L. Gintsburg

**Affiliations:** ^1^Department of Allergy and Immunology, City Clinical Hospital No.52 of Moscow Healthcare Department, Moscow, Russia; ^2^State Virus Collection Laboratory, Federal State Budget Institution “National Research Centre for Epidemiology and Microbiology named after Honorary Academician N F Gamaleya” of the Ministry of Health of the Russian Federation, Moscow, Russia; ^3^Allergy and Immunology Department, Federal State Autonomous Educational Institution of Higher Education I.M. Sechenov First Moscow State Medical University of the Ministry of Health of the Russian Federation (Sechenov University), Moscow, Russia; ^4^General Therapy Department, Pirogov Russian National Research Medical University, Moscow, Russia

**Keywords:** neutralizing antibodies, Tixagevimab/Cilgavimab, Regdanvimab, COVID-19, omicron

## Abstract

Several virus-neutralizing monoclonal antibodies (mAbs) have become new tools in the treatment of the coronavirus disease (COVID-19), but their effectiveness against the rapidly mutating virus is questionable. The present study investigated the effectiveness of Tixagevimab/Cilgavimab and Regdanvimab for mild and moderate COVID-19 treatment in real-world clinical practice during the Omicron variant-dominant period. Patients with known risk factors for disease progression and increasing disease severity were enrolled in the study within the first 7 days of symptom onset. Seventy-seven patients were divided into four groups: first 15 patients received 300 mg Tixagevimab/Cilgavimab intravenously (IV) and 23 patients got the same drug 300 mg intramuscularly (IM), the next 15 patients was on the same combination in dose of 600 mg IV, and 24 patients were on Regdanvimab at a dose of 40 mg/kg IV. By Day 4, 100% of Tixagevimab/Cilgavimab IV patients showed negative polymerase chain reaction results for SARS-CoV-2 Ribonucleic acid (RNA) regardless of the mAbs dose while in the Regdanvimab group 29% of the patients were positive for SARS-CoV-2 virus RNA. The testing for virus neutralizing antibodies (nAbs) to various Omicron sublineages (BA.1, BA.2, and BA.5) showed that an increase in nAb levels was detected in blood serum immediately after the drug administration only in Tixagevimab/Cilgavimab 300 mg and 600 mg IV groups. In the group of intravenous Regdanvimab, a significant increase in the level of nAbs to the Wuhan variant was detected immediately after the drug administration, while no increase in nAbs to different Omicron sublineages was observed.

**Clinical trial registration:**

https://clinicaltrials.gov/, identifier NCT05982704.

## 1 Introduction

Vaccination against the new coronavirus infection COVID-19 has reduced morbidity, mortality and the burden on the healthcare system worldwide (1). However, there is a cohort of patients with reduced immune response, including that to active immunization (2). In some patients this may be due to the development of primary (3) and secondary immunodeficiency conditions associated, for example, with the use of immunosuppressants (4–8). At the same time, the immune response can be also affected by different factors. There is evidence of age-related dysregulation and reduced immunity (immunosenescence) (9). For people aged 50–64 years the risk of COVID-19-associated death increases fourfold or more compared with people younger than 40 years, and such increase is more than tenfold for people over 85 years of age (10). The presence of chronic diseases plays a major role, where beside comorbidity itself concomitant therapy and the age of the patient play an important role. The risks of death in those with one comorbidity and more than 10 comorbidities are respectively 1.5 and 3.8 times greater than in people without comorbidities (11–13). There are a number of conditions that particularly reduce the immune response to vaccination: hemodialysis due to terminal renal failure (14), diabetes mellitus (15), lymphoproliferative disorders (16) and others. Even sex due to the influence of sex-related hormones (e.g. estrogen vs. testosterone) influences vaccine response (17).

We have observed a revolution in the treatment of coronavirus infection and development of passive immunity against it. Nowadays several SARS-CoV-2-specific neutralizing mAbs in use possess not only a direct antiviral effect, but also the capability for prolonged circulation underpinning their protective potential (18). The use of various mAbs to treat the coronavirus infection was approved by the US Food and Drug Administration (FDA) in 2020–2021 (19–21). The mAb spectrum includes both single component (sotrovimab, regdanvimab) and combination drugs (bamlavimab/etesivimab, casirivimab/imdevimab, cilgavimab/tixagevimab). The mAbs have shown their efficacy and safety both in clinical trials and in real clinical practice, especially in immunocompromized patients (22, 23).

SARS-CoV-2 actively acquires new mutations leading to the appearance of numerous new SARS-CoV-2 variants (23). The emergence of mutations in the SARS-CoV-2 spike glycoprotein may critically reduce the efficacy of the mAbs-based anti-COVID-19 therapies and antiviral drugs (24). The reduction of anti-spike monoclonal antibody effectivenes has previously been shown with the Delta strain (25). Nowadays one such variant is Omicron. Shortly after its identification Omicron was designated by WHO as a “variant of concern” (VOC), and to date it remains the only VOC. The pronounced rise of COVID-19 incidence was detected worldwide since the Omicron variant surge (26). It was also noted that currently endemic Omicron sublineages BA.4 and BA.5 have the impact on the integrity and sensitivity of reverse-transcription polymerase chain reaction (RT-qPCR) assays used for COVID-19 diagnosis (27).

The mAbs have demonstrated efficacy and safety in many COVID-19 clinical trials around the world (28–33). However, the emergence of new SARS-CoV-2 variants underscores the need for continuous monitoring of the effectiveness of mAbs (26, 34).

Tixagevimab/Cilgavimab was one of the first mAbs registered worldwide for COVID-19 treatment, including the Russian Federation (35). This drug is a combination of two fully humanized monoclonal antibodies isolated from B cells of individuals with COVID-19. These antibodies recognize non-overlapping regions of the receptor-binding domain (RBD) of SARS-CoV-2 glycoprotein S (33).

Due to the generalized spread of different SARS-CoV-2 Omicron sublineages, we conducted a study of the effectiveness of virus-neutralizing monoclonal antibodies (Tixagevimab/Cilgavimab [Evusheld] and Regdanvimab [Regkiron]) for the treatment of the new coronavirus infection (COVID-19) in adult patients.

## 2 Materials and methods

### 2.1 Study design and participants

We conducted a non-randomized, single-center, prospective observational cohort study. The study was performed between August 20, 2022 and February 1, 2023 within the facilities of the multidisciplinary City Clinical Hospital No. 52 (Moscow, Russian Federation) and the N.F. Gamaleya Research Institute of Epidemiology and Microbiology (Moscow, Russian Federation). The trial was conducted in accordance with the ethical principles derived from international guidelines, including the Declaration of Helsinki, and was approved by the local ethics committee (version 1.1 of 08.09.2022). The study was registered on ClinicalTrials.gov (ID NCT05982704).

We included adults (18 years or older) of both sexes according to the following inclusion and exclusion criteria. Inclusion criteria were: 1) confirmed diagnosis of a new mild or moderate COVID-19 coronavirus infection (36), 2) manifestation of COVID-19 symptoms within 7 days prior to the inclusion, 3) known risk factors for the progression and severe course of COVID-19 (36). Exclusion criteria were: 1) hypersensitivity to the active substance or other excipients (for the Evusheld group: histidine, histidine hydrochloride monohydrate, sucrose, polysorbate 80, methionine; for the Regkiron group: L-histidine, L-histidine monohydrochloride monohydrate, polysorbate 80, L-arginine monohydrochloride), 2) history of anaphylactic reactions to mAbs, 3) need for oxygen therapy at the time of inclusion in the study, 4) pregnancy or breastfeeding. All participants gave informed consent to participate in the study, as well as to the administration of the drug Tixagevimab/Cilgavimab or Regdanvimab.

### 2.2 Description of medical intervention

**Figure 1:**
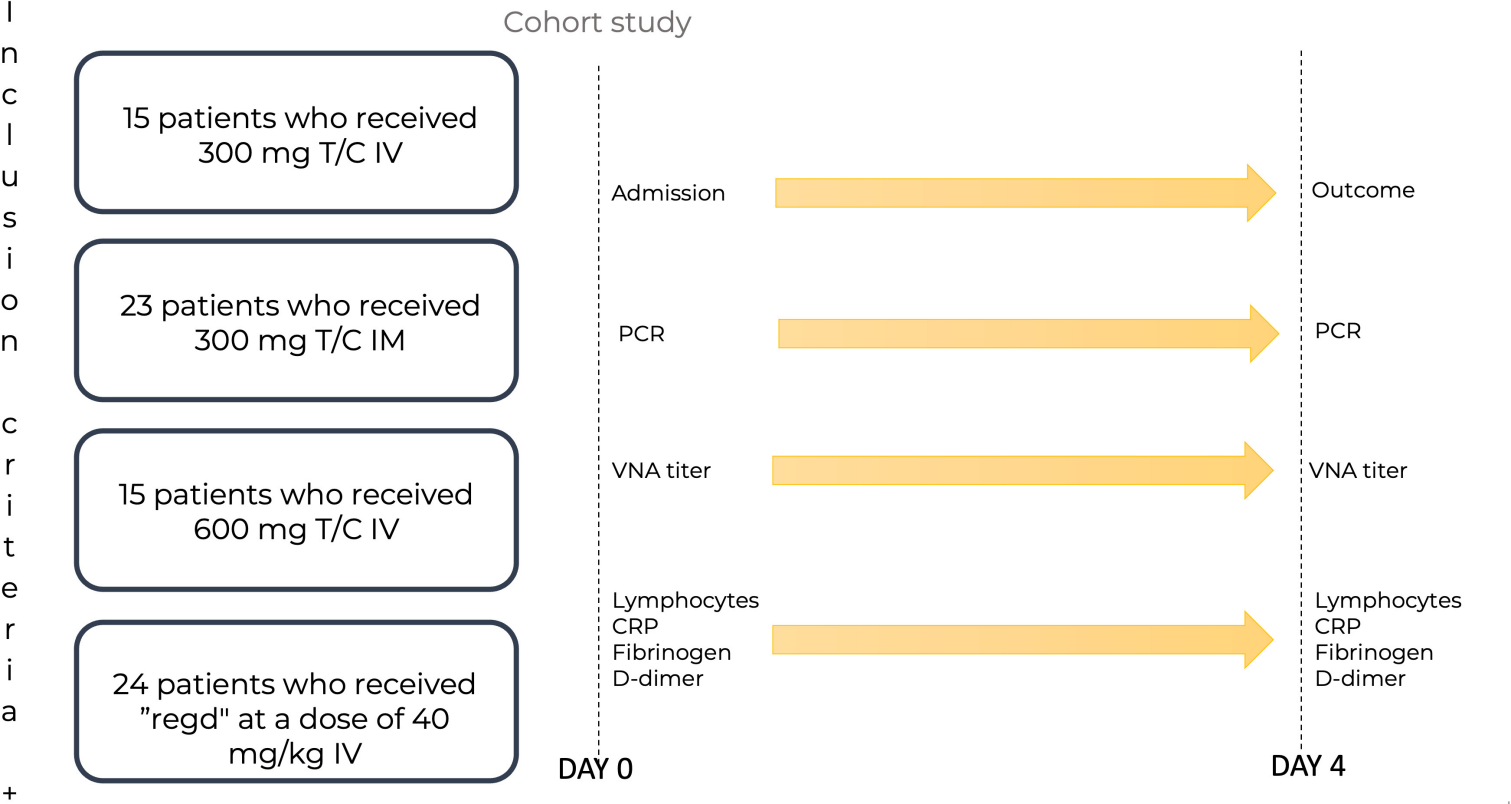
The scheme of patient recruitment for the study. T/C, Tixagevimab/Cilgavimab; PCR, polymerase chain reaction; IM, intamuscularly; IV, intravenously; mg, milligram; kg, kilogram; Regd, Regdanvimab; VNA, virus neutralizing antibody; CRP, C-reactive protein. Patients were admitted to the Day patient department. The patients were divided into four groups: 15 patients who received Tixagevimab/Cilgavimab IV 300 mg; 23 patients who received Tixagevimab/Cilgavimab IM 300 mg; 15 patients who received Tixagevimab/Cilgavimab IV 600 mg; 24 patients who received Regdanvimab IV at a dose of 40 mg/kg of body weight. PCR, VNA titer, lymphocyte count, PCR, fibrinogen, and D-dimer were monitored on day 0. On day 4, all the above parameters were monitored, and the outcomes were recorded.

The study included 77 patients diagnosed with COVID-19 as outpatients by qualitative determination of the SARS-CoV-2 antigen or SARS-CoV-2 RNA, with mild or moderate disease course and a high risk of disease progression. The severity of coronavirus infection and the risks were determined according to the Russian Interim Clinical Guidelines on COVID-19 (36). The mAbs were administered in the day patient facility and in some cases in the hospital inpatient department. The scheme for recruiting patients into groups is shown in **Figure 1**.

The patients were divided into four groups:

1. 15 patients who received Tixagevimab/Cilgavimab IV 300 mg.
2. 23 patients who received Tixagevimab/Cilgavimab IM 300 mg.
3. 15 patients who received Tixagevimab/Cilgavimab IV 600 mg.
4. 24 patients who received Regdanvimab IV at a dose of 40 mg/kg of body weight.

No randomization was performed as it was a real world clinical study.

The administration of drugs in all groups of patients was regulated by the Russian Interim Clinical Guidelines on COVID-19 (36). Patients also received additional anti-inflammatory therapy (interleukin-1-R and -6-R inhibitors) according to indications. The use of anti-inflammatory therapy did not differ between the groups.

Immediately before the drug administration (Day 0) as part of routine clinical practice to assess the severity of COVID-19 all patients underwent the following tests and procedures: full blood count, biochemical blood assay (including CRP and lactate dehydrogenase [LDH]), coagulogram (including fibrinogen and D-dimer), chest СТ scan, ECG, nasopharyngeal swab for polymerase chain reaction (PCR) testing to detect SARS-CoV-2 RNA. Additionally, after signing the informed consent to participate in the study the patients also underwent blood serum sampling for the test of virus neutralization activity before the administration of mAbs and an hour after the administration of mAbs. On Day 4 after the administration of Tixagevimab/Cilgavimab or Regdanvimab the patients underwent the following tests and procedures: full blood count, biochemical blood assay (including CRP and LDH), coagulogram (including fibrinogen and D-dimer), chest СТ scan, ECG, nasopharyngeal swab for PCR testing to detect SARS-CoV-2 RNA and blood serum testing for virus neutralization activity.

### 2.3 Neutralization assay

Investigations of live SARS-CoV-2 viruses were performed in BSL-3 facilities. We used the following SARS-CoV-2 sublineages for neutralization assays: B.1.1.1 (Wuhan, S:D614G hCoV-19/Russia/Moscow_PMVL-1/2020), B.1.1.529 Omicron BA.1 (hCoV-19/Russia/MOW-Moscow_PMVL-O16/2021), B.1.1.529 Omicron BA.2 (hCoV-19/Russia/MOW-PMVL-ON402/2022), B.1.1.529 Omicron BA.5 (hCoV-19/Russia/SPE-RII-25357S/2022). Viruses were propagated and titrated in Vero E6 cells. Viruses were titrated by microtitration method; titers were determined by the 50% tissue culture infective dose (TCID50) method, the titer was determined by the Spearman–Kaerber method. Determination of the neutralizing antibody levels in serum samples was performed by the microneutralization test as described earlier (21). Briefly, blood serum samples were inactivated at 56°C for 30 minutes, then serum dilutions were prepared in DMEM culture medium with 2% inactivated fetal bovine serum, 50 µl of serum dilutions were mixed with 100 TCID50 of the SARS-CoV-2 virus (50 µl), incubated for 1 hour at 37°C and added to Vero E6 cells in 96-well plates. The cells were incubated at 37°C in 5% CO_2_; after 96 hours, the development of cytopathic effect of the virus on the cell culture was recorded visually. The titer of the virus neutralization activity of the studied serum was reported as the highest dilution at which the cytopathic effect was suppressed.

### 2.4 Outcomes

We evaluated the laboratory efficacy, clinical results and titers of virus neutralizing antibodies (nAbs) against the Wuhan variant and Omicron sublineages BA.1, BA.2, BA.5. Primary endpoints included decrease of the positive SARS-CoV-2 PCR results on Day 4 and the nAbs increase after administration one hour and on Day 4 after administration in comparison with Day 0 (i.e. before mAbs administration).

The secondary endpoints in the study included evaluation of lymphocyte concentration changes, measurement of markers of systemic inflammation (CRP, fibrinogen, D-dimer) on Day 4 of observation, and the outcome of the disease (discharge, hospitalization, death).

### 2.5 Statistical analysis

Principles for calculating the sample size: no preliminary calculation of the required sample size was carried out. Statistical data analysis: nonparametric methods of descriptive statistics were used. The median and interquartile range (IQR) were determined; the geometric mean was used in the description of relative values over time. The data analysis was performed using the IBM SPSS STATISTICS v.22 statistical program package. To compare quantitative data, the Mann–Whitney U-test and the Kruskal–Wallis test were used depending on the number of groups being compared; Pearson’s χ2 test was used for categorical data. Spearman’s rank correlation coefficient was used to compare nonlinear indicators. The differences were considered significant at p<0.05.

## 3 Results

### 3.1 Description of patients and laboratory indicators

**Figure 2:**
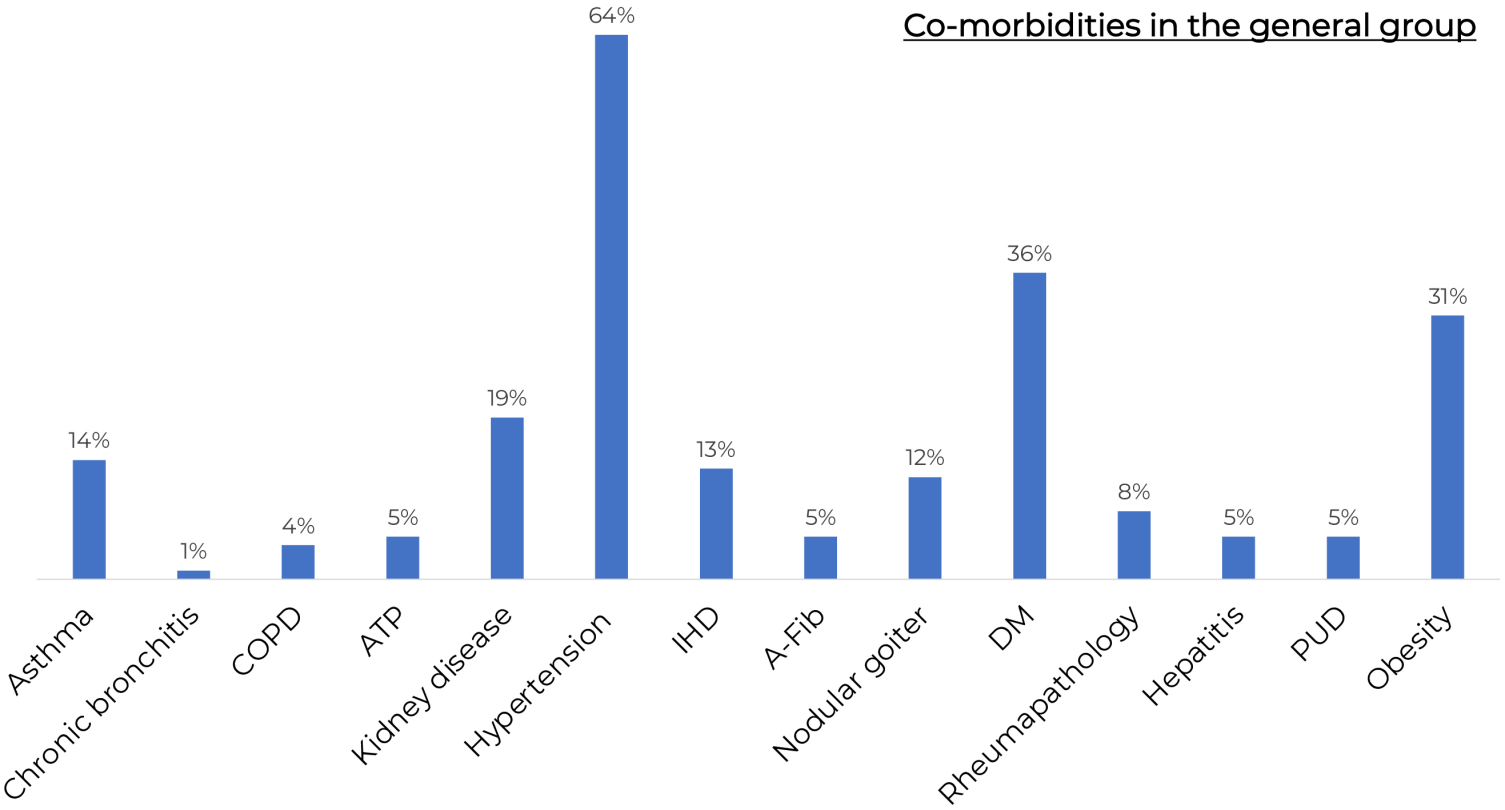
The frequency of comorbidities in the general group of patients. COPD, Chronic obstructive pulmonary disease; KAT, kidney allotransplantation; IHD, ischaemic heart disease; A-Fib, atrial fibrillation; DM, Diabetes Mellitus; GUD, Gastric ulcer disease. All groups had high levels of comorbidity.

The general characteristics of COVID-19 patients admitted to the day patient facility for antiviral therapy are presented in **Table 1**. There were no significant differences in age, gender, COVID-19 and/or vaccination against COVID-19 history between the patients of different virus-neutralizing therapy groups. Most of the patients were admitted to the day patient facility on Day 3 after the onset of symptoms. During the observation period the condition of the patients in all groups was stable, and the body temperature was in the normal range. All groups had high levels of comorbidity (**Figure 2**); the median age of patients was 63 years (IQR 53–71 years).

**Table 1:** General patient data.

Data	Tixagevimab/cilgavimab 150 + 150 mg IV (n=15)	Tixagevimab/cilgavimab 150 + 150 mg IM (n=23)	Tixagevimab/cilgavimab 300 + 300 mg IV (n=15)	Regdanvimab 40 mg/kg body weight (n=24)	p
SEX					0,164
F M	9 (60%) 6 (40%)	17 (74%) 6 (26%)	9 (60%) 6 (40%)	20 (83%) 4 (17%)	
COVID-19 pneumonia	4 (27%)/ n=15	3 (13%)/ n=23	1 (7%)/ n=15	3 (13%)/ n=24	0,444
History of COVID-19	5 (36%)/ n=14	8 (36%)/ n=22	5 (36%)/ n=14	3 (12.5%)/ n=24	0,278
COVID-19 vaccination	3 (23%)/ n=13	12 (52%)/ n=23	7 (50%)/ n=14	11 (46%)/ n=24	0,242
Age	69 (60-80)/n=15	60 (56-68)/n=23	63 (39.5-68.8)/n=15	63.5 (51.25-70)/n=24	0,082
Day of disease on admission	2 (2-7)/ n=15	3 (3-4)/ n=23	3 (2-4.8)/ n=15	3 (3-4.75)/ n=24	0,814
BMI	26.8 (23.8-29)/n=15	26.6 (21.4-34.5)/n=23	24.35 (21.6-30.43)/n=15	25.9 (24- 31.7)/n=24	0,79
Maximum temperature	38.5 (37.1-39)/n=15	38.7 (38.5-39)/n=23	38.4 (37.8-38.9)/n=15	38.5 (37.9-38.9)/n=21	0,308
Temperature on day 4 of the study	36.6 (36.5-36.6)/n=14	36.4 (36.4-36.5)/n=22	36.45 (36.2-36.6)/n=16	36.6 (36.2-36.6)/n=23	0,8

IV, intravenously; IM, intramuscularly; F, female; M, male; BMI, body mass index; mg, milligram, kg, kilogram.

Quantitative data are presented as median and interquartile range (Q1-Q3).

All patients were vaccinated with Gam-COVID-Vac. The frequency of vaccination was not significantly different between groups (p = 0.5). The duration between vaccine and mab administration was also not significantly different between groups.

Most of the patients were admitted to the day patient facility on Day 3 after the onset of symptoms. During the observation period the condition of the patients in all groups was stable, and the body temperature was in the normal range.

**Figure 3:**
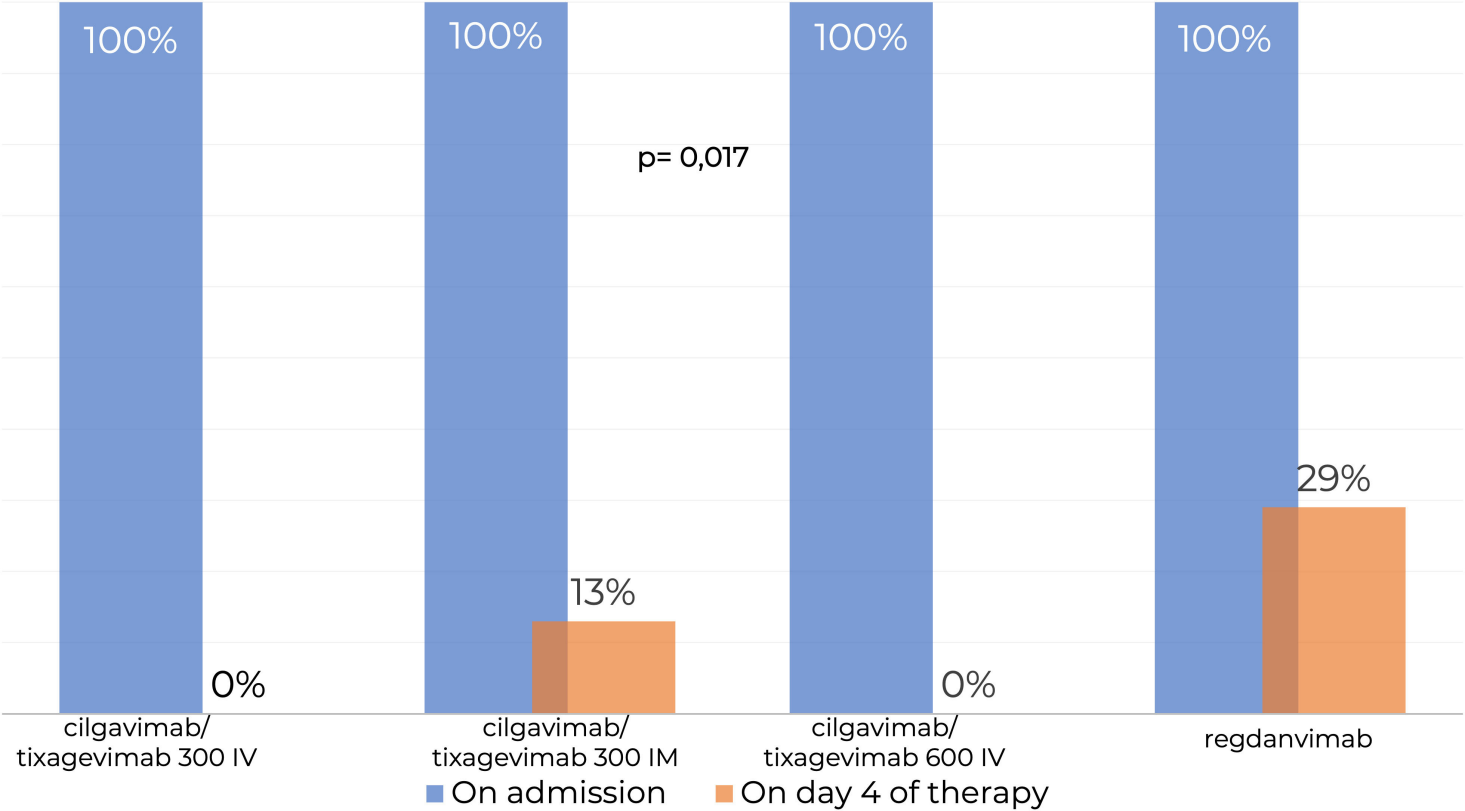
Testing for SARS-CoV-2 RNA by real-time PCR in nasopharyngeal swabs. IM, intamuscularly; IV, intravenously. SARS-CoV-2 RNA PCR testing showed that 100% patients in the Tixagevimab/Cilgavimab IV groups tested negative at Day 4 of the study regardless of the dose, while 29% of patients in the Regdanvimab group tested positive for SARS-CoV-2 RNA, which was significantly higher.

SARS-CoV-2 RNA PCR testing showed that 100% patients in the Tixagevimab/Cilgavimab IV groups tested negative at Day 4 of the study regardless of the dose, while 29% of patients in the Regdanvimab group tested positive for SARS-CoV-2 RNA, which was significantly higher (p=0,017) (**Figure 3**).

### 3.2 Levels of virus-neutralizing antibodies

The study was conducted during the surge of the SARS-CoV-2 Omicron BA.5 sublineage. Genetic analyses of the patients’ nasopharyngeal swabs by real-time PCR confirmed that all swabs contained SARS-CoV-2 Omicron sublineage BA.5 RNA.

We determined the levels of nAbs to the initial SARS-CoV-2 variant (Wuhan D614G) and to the Omicron sublineages BA.1, BA.2 and BA.5 in the blood serum of the patients to identify the neutralization activity of nAbs. In the IV treatment groups (Tixagevimab/Cilgavimab 300 mg, Tixagevimab/Cilgavimab 600 mg and Regdanvimab groups), we observed a strong increase in nAbs to the Wuhan variant immediately after the drug administration (**Table 2**), with the highest nAbs levels detected in the Regdanvimab group. However, the investigation of nAbs against different Omicron sublineages (BA.1, BA.2, and BA.5) showed an increase in nAbs immediately after drug administration in the Tixagevimab/Cilgavimab IV 300 mg and 600 mg groups only. In the Regdanvimab group, an increase in nAbs to different Omicron sublineages was detected only on Day 4 after drug administration, which indicated the development of immune response over the course of the disease (**Table 2**).

**Table 2:** Titers mAbs to different SARS-CoV-2 variants.

Virus strain	Time point	Tixagevimab/cilgavimab 150 + 150 mg IV		Tixagevimab/cilgavimab 150 + 150 mg IM		Tixagevimab/cilgavimab 300 + 300 mg IV		Regdanvimab 40 mg/kg body weight		p
		GMT	95% CI	GMT	95% CI	GMT	95% CI	GMT	95% CI	
Wuhan	before	80	10.15-630.6	28.28	7.80-102.6	9.17	2.77-3.38	36.68	12.69-106.1	0,247
	after	13116	8507-20223	127	38.44-419.5	10240	2911-36027	715897	395686-1295240	<0,001
	Day 4	10240	7397-14176	3763	2155-6569	11763	3488-39667	242419	150496-390489	<0,001
Omicron ВА.1	before	12.81	3.08-53.35	7.937	3.86-16.31	6.771	3.16-14.52	5.612	2.26-13.91	0,57
	after	118.9	66.47-212.6	10.29	4.75-22.33	73.36	43.61-123.4	6.174	2.49-15.34	<0,001
	Day 4	127	47.58-339	209.5	61.62-712.3	1341	446.3-4026	50.91	15.53-166.7	0,00§
Omicron ВА.2	before	16.41	4.7-57.28	7.071	3.56-14.03	12.42	4.16-37.06	6.674	2.657-16.77	0,287
	after	861.4	528.1-1405	10.29	4.63-22.86	2248	966-5231	6.965	2.89-16.8	<0,001
	Day 4	1076	592.2-1956	615.8	317.3-1195	1222	399.3-3741	68.81	25.89-182.9	<0,001
Omicron ВА.5	before	24.38	8.18-72.69	7.711	3.89-15.28	3.692	1.83-7.44	11.89	5.49-25.77	0,025
	after	176.7	110.7-281.8	11.55	5.79-23.04	59.07	38.59-90.43	13.93	6.44-30.13	<0,001

IV, intravenously; IM, intramuscularly; mg, milligram, kg, kilogram; GMT, geometric mean titer; CI, confidence interval.

Interestingly, in the Tixagevimab/Cilgavimab IM 300 mg group the levels of nAbs to the Omicron sublineages reached its maximum by Day 4 of the study, with values significantly higher than those in the Regdanvimab group (also on Day 4). It is important to note that the levels of nAbs to different Omicron sublineages did not differ significantly on Day 4 between the groups of IV and IM administration of Tixagevimab/Cilgavimab at a dose of 300 mg.

Analysis of the nAb levels to different SARS-CoV-2 sublineages over time within each group revealed the following patterns:

1. Tixagevimab/Cilgavimab IV administration groups: a significant increase in the levels of nAbs to the Wuhan and Omicron BA.1 and BA.2 sublineages was detected immediately after the drug administration, the nAb levels remained stable for 4 days; a significant increase in the levels of nAbs to the Omicron BA.5 sublineage was detected immediately after drug administration, and then the nAb levels increased over time and reached their maximum on Day 4 of the study, which indicated the development of the host immune response.
2. Tixagevimab/Cilgavimab IM administration group: the highest nAb levels were detected on Day 4 of the study. At the same time, the levels of nAbs to the Omicron sublineages on Day 4 of the study did not differ between the groups of IV and IM administration.
3. Regdanvimab IV administration group: a significant increase in the levels of nAbs to the Wuhan variant was detected immediately after administration, while there was no increase in nAbs to different sublineages of the Omicron variant. On Day 4 of the study, a decrease in nAbs to the Wuhan variant was detected, indicating the removal of nAbs from the systemic circulation. The increase in nAbs to the Omicron sublineages on day 4 of the study indicates the development of immune response.
4. In the groups that received Tixagevimab/Cilgavimab the levels of nAbs to the Omicron sublineage BA.5 on Day 4 of the study were significantly higher (about 10 times) than in the Regdanvimab group.

**Figure 4:**
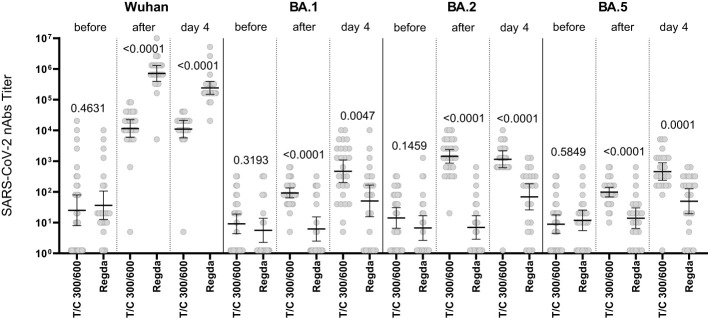
Comparison of nAbs levels in the IV Tixagevimab/Cilgavimab and IV Regdanvimab patient groups. T/C, Tixagevimab/Cilgavimab; Regda, Regdanvimab. The levels of nAbs to the Omicron BA.1, BA.2 and BA.5 sublineages in the intravenous Tixagevimab/Cilgavimab group were significantly higher than those in the Regdanvimab group. A robust increase in nAbs to the Wuhan variant of SARS-CoV-2 virus was detected in patients who received Regdanvimab immediately after administration, while no increase of nAb levels to the Omicron BA.1, BA.2 and BA.5 sublineages was seen.

The levels of nAbs to the Omicron BA.1, BA.2 and BA.5 sublineages in the intravenous Tixagevimab/Cilgavimab group were significantly higher than those in the Regdanvimab group. A robust increase in nAbs to the Wuhan variant of SARS-CoV-2 virus was detected in patients who received Regdanvimab immediately after administration, while no increase of nAb levels to the Omicron BA.1, BA.2 and BA.5 sublineages was seen (**Figure 4**).

**Figure 5:**
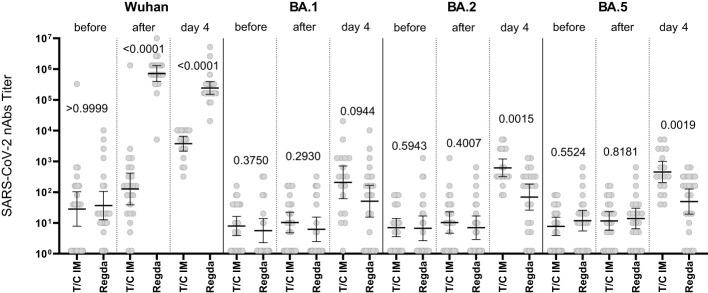
Comparison of nAbs levels in IM Tixagevimab/Cilgavimab and Regdanvimab patient groups. T/C, Tixagevimab/Cilgavimab; Regda, Regdanvimab; IM, intramuscularly. The levels of nAbs to Omicron BA.2 and BA.5 sublineages in the Tixagevimab/Cilgavimab IM group were significantly higher by Day 4 than in the Regdanvimab group.

The nAb titer for the Wuhan variant in patients who received Tixagevimab/Cilgavimab IV exceeded 10,000; the nAb titer for the Omicron BA.5 sublineage Tixagevimab/Cilgavimab group on Day 4 of the study was 450, with a greater than 22-fold decrease in the neutralization activity when compared to the Wuhan variant. The levels of nAbs to Omicron BA.2 and BA.5 sublineages in the Tixagevimab/Cilgavimab IM group were significantly higher by Day 4 than in the Regdanvimab group (**Figure 5**).

**Figure 6:**
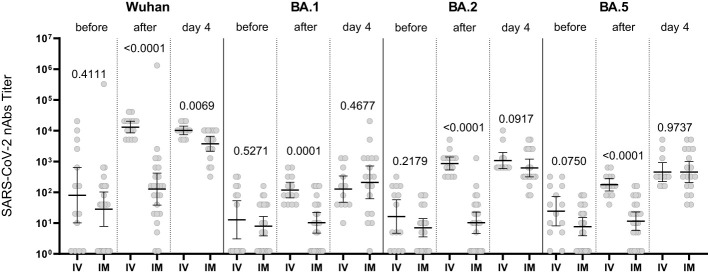
Comparison of nAbs levels in IV and IM Tixagevimab/Cilgavimab patient groups. IM, intamuscularly, IV, intravenously. The increase in the nAb titer in the blood serum of patients who received Tixagevimab/Cilgavimab IM occurred with a delay (compared to IV administration). By Day 4 of observation the levels of nAbs to Omicron BA.1, BA.2, BA.5 sublineages did not differ from the Tixagevimab/Cilgavimab IV group.

The increase in the nAb titer in the blood serum of patients who received Tixagevimab/Cilgavimab IM occurred with a delay (compared to IV administration). However, by Day 4 of observation the levels of nAbs to Omicron BA.1, BA.2, BA.5 sublineages did not differ from the Tixagevimab/Cilgavimab IV group (**Figure 6**).

**Figure 7:**
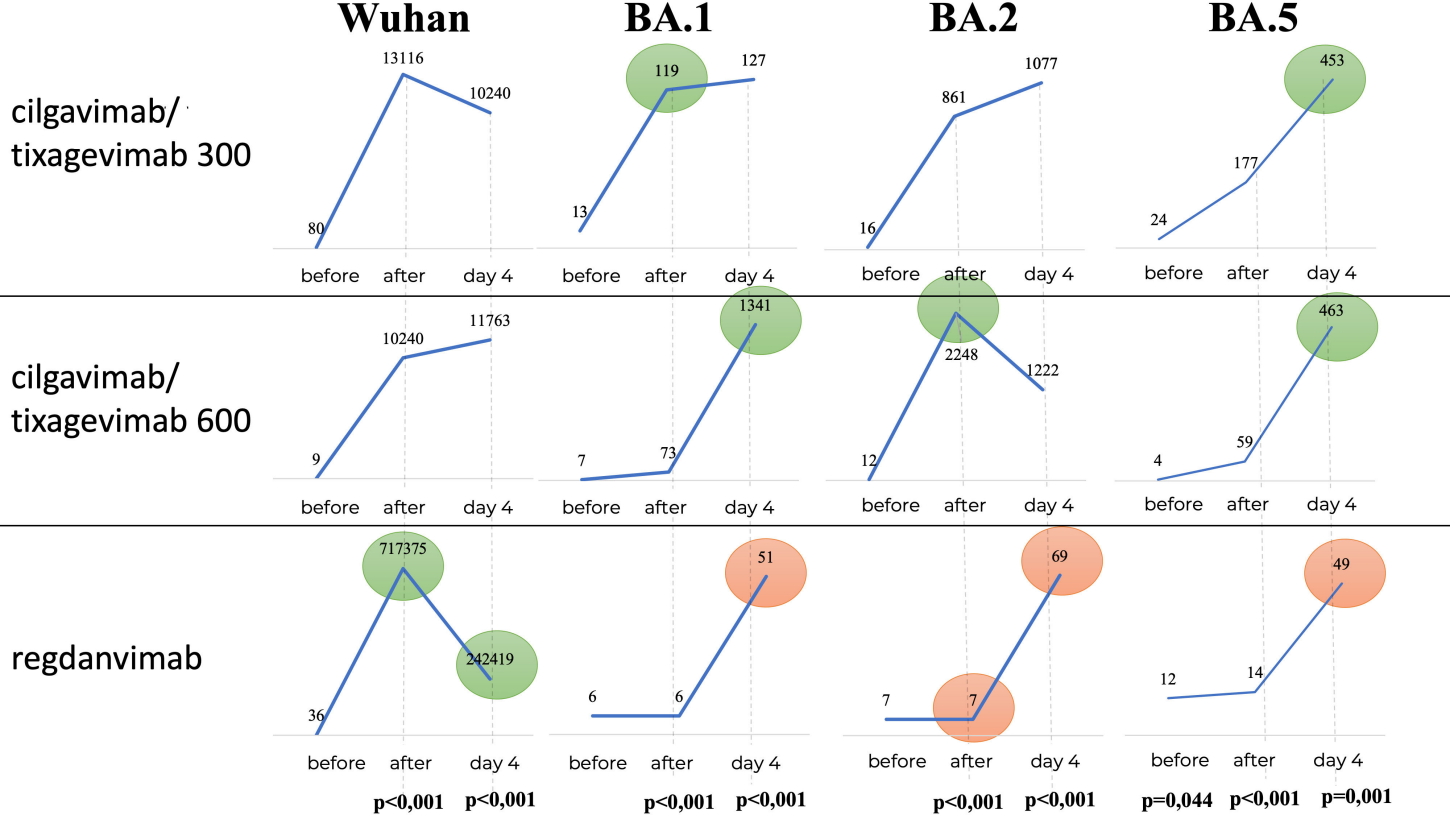
Geometric mean of nAbs titers to different variants of SARS-CoV-2. In the Regdanvimab group, the geometric mean of nAb titers to the Wuhan variant was higher than that in the Tixagevimab/Cilgavimab groups, both one hour after administration and on Day 4 (p<0.001), while an inverse effect was observed for the concentrations of nAbs against Omicron sublineages (p<0.001).

**Figure 7** shows that in the Regdanvimab group, the geometric mean of nAb titers to the Wuhan variant was higher than that in the Tixagevimab/Cilgavimab groups, both one hour after administration and on Day 4 (p<0.001), while an inverse effect was observed for the concentrations of nAbs against Omicron sublineages (p<0.001).

When analyzing the outcome of the disease as a secondary endpoint, 100% recovery was observed in the Tixagevimab/Cilgavimab groups regardless of the route of administration. In the Regdanvimab group, 2 patients (8.3%) were transferred from the day patient facility to the hospital inpatient department due to the appearance of new foci of ground-glass opacities seen on chest CT, as well as the absence of positive laboratory dynamics. However, the difference between the groups was not statistically significant (p =0,209) (**Table 3**).

**Table 3:** Outcomes of the disease.

Feature	Tixagevimab/Cilgavimab 300 mg IM	Tixagevimab/Сilgavimab 300 mg IV	Tixagevimab/Cilgavimab 600 mg IV	Regdanvimab 40 mg/kg body weight	p
Recovery Transfer to the hospital	15/15; 100% 0; 0%	23/23; 100% 0; 0%	15/15; 100% 0; 0%	24/26; 92% 2/26; 8%	0,209

IM, intamuscularly; IV, intravenously; mg, milligram; kg, kilogram.

**Figure 8:**
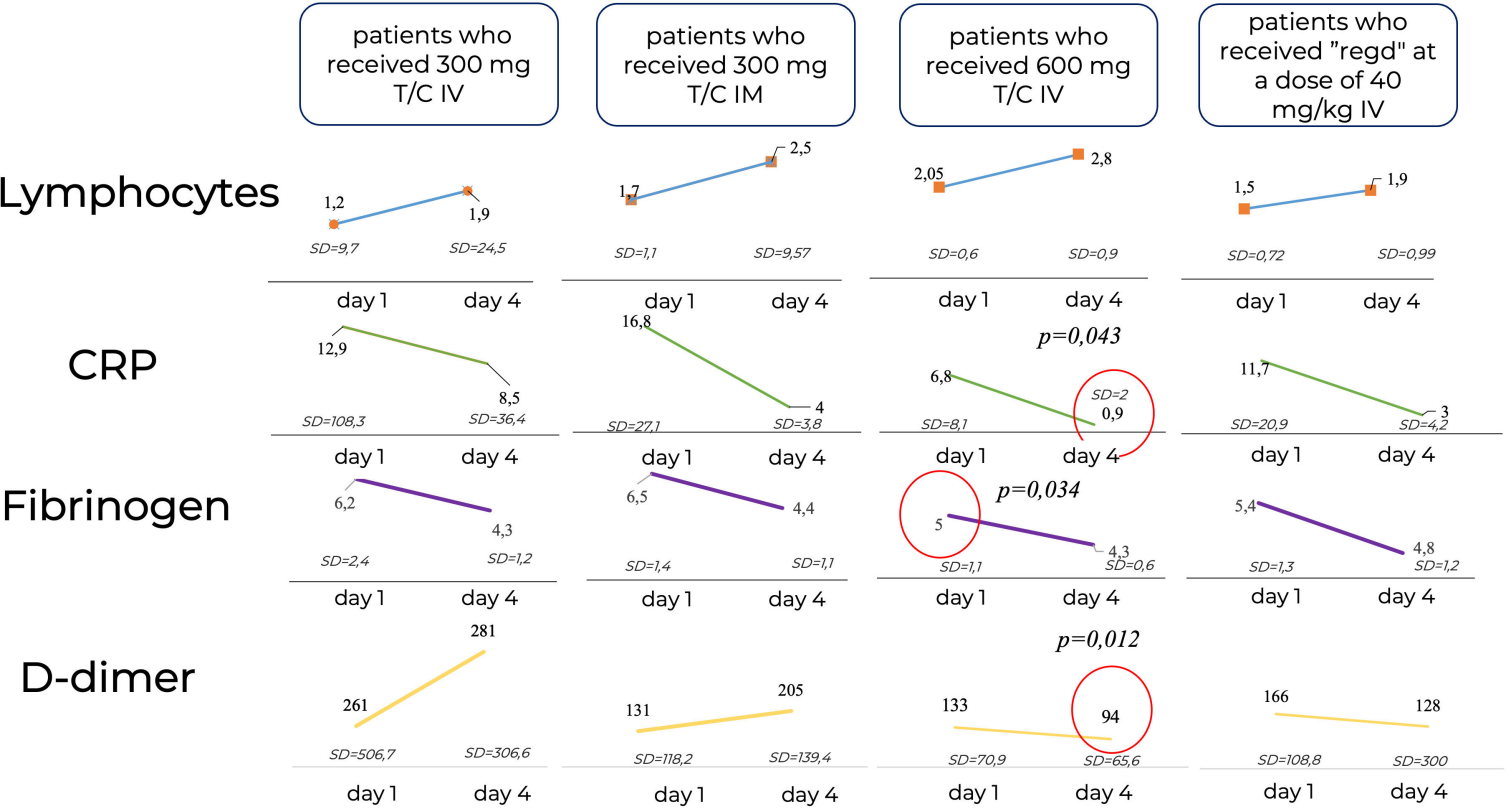
Laboratory parameters over time. CRP, C-reactive protein; IM, intamuscularly, IV, intravenously, mg, milligram, kg, kilogram; Regd, Regdanvimab. The lymphocyte levels did not differ significantly between patients, while the levels of CRP and D-Dimer were significantly lower in the Tixagevimab/Cilgavimab 300 + 300 mg IV group by Day 4 of the observation than in the other groups regardless of the ongoing concomitant anti-inflammation therapy.

The study of laboratory parameters over time showed that the lymphocyte levels did not differ significantly between patients, while the levels of CRP and D-Dimer were significantly lower in the Tixagevimab/Cilgavimab 300 + 300 mg IV group by Day 4 of the observation than in the other groups regardless of the ongoing concomitant anti-inflammation therapy (**Figure 8**).

## 4 Discussion

During COVID-19 pandemic, SARS-CoV-2 was rapidly acquiring new mutations with the wide spread of new variants among the world population. At the start of our research in September 2022, the Omicron variant consisted of 5 main sublineages, BA.1, BA.2, BA.3, BA.4, and BA.5 (37). It was shown that, unlike other variants, Omicron was characterized by the complete change in the antigenicity of the S protein (38). The new Omicron sublineages BA.1 (B.1.1.529.1) and BA.2 (B.1.1.529.2) had higher transmission rates than the previous variants, including Delta (B.1.617.2), as well as a large number of mutations within the glycoprotein, especially in the RBD (39). For example, sublineage BA.1 has 34 mutations in S protein, BA.2 —30 mutations, BA.4 and BA.5 differ from strain BA.2 by having 3 mutations in the RBD and one deletion in the N-terminal domain (40). Monoclonal antibodies used in clinical practice predominantly target the RBD, thus many mAbs have shown reduced activity against the Omicron sublineages or no activity at all in the neutralization assay (37).

According to our previous study from February 2022, when the epidemiological data suggested the beginning of the Omicron surge, the use of mAb Regdanvimab led to a significant decrease in the severity of clinical manifestations according to the Clinical Progression Scale (41). Negative PCR results were observed in 82% of the patients on Day 4 after the drug administration. One disadvantage of the previous study was the lack of virus typing, which casts doubt on the exact variant distribution. The results of the present study demonstrate that Regdanvimab, which was effective against the Wuhan variant, lost it’s virus neutralization activity against the BA.1, BA.2, and BA.5 sublineages of Omicron. No increase in nAbs against Omicron sublineages was observed one hour after administration. A slight increase in nAbs to Omicron sublineages by Day 4 after the administration may be due to the immune response development during the active disease. This was reflected in the persistent positive PCR results in 29% of cases in the Regdanvimab group on Day 4 when compared with the other groups (p<0.017). In addition, in two Regdanvimab-treated patients (8.3%) the disease progressed with new lesions seen on chest CT.

Despite the persistent virus-neutralising activity of tixagevimab/cilgavimab against the Omicron variant, it was still reduced by 22-fold compared to the Wuhan variant. The decrease of virus-neutralizing activity against Omicron is confirmed by literature data. Cao et al. reported that the activity of Bamlanivimab/Etesevimab, Casirivimab/Imdevimab, and Tixagevimab/Cilgavimab was significantly weakened against BA.2.12.1, BA.4, and BA.5 Omicron sublineages, while Sotrovimab was still active, but with reduced effectiveness (42). In a further study they clarified that Cilgavimab retained it’s neutralizing activity against the BA.2.12.1 (IC50 ≤ 30 ng/mL) and BA.4/BA.5 (30 ng/mL < IC50 < 1,000 ng/mL) Omicron sublineages (42). In the study by Hu et al. Cilgavimab from the Tixagevimab/Cilgavimab combination was the only mAb to retain it’s virus-neutralizing activity against Omicron (43). According to Boschi et al., only the second mAb from the combination of Tixagevimab/Cilgavimab retained it’s activity against the Omicron variant as well (44). At the same time, Tixagevimab/Cilgavimab (EC50 = 1.185 and 369 ng/mL, respectively) demonstrated successful *in vitro* neutralization of SARS-CoV-2 variants, including Omicron, while maintaining activity against BA.1 and BA.2 sublineages (38). According to the data obtained in our present study, the neutralizing activity in patients treated with Tixagevimab/Cilgavimab against BA.1, BA.2, and BA.5 sublineages was significantly higher than that of Regdanvimab regardless of the dose of the administered drug. A higher concentration of nAbs was observed on Day 4 after administration of Tixagevimab/Cilgavimab rather than one hour after administration (except for the concentration of nAbs to the BA.2 sublineage after the administration of Tixagevimab/Cilgavimab 600 mg). The *in vitro* study by Roe et al. also demonstrated a significant drop in the virus-neutralizing activity of Tixagevimab/Cilgavimab when compared to that against the Omicron BA.1 sublineage (IC50 1.5 ng/mL versus 389.2 ng/mL, respectively) (45). Bruel et al. demonstrated that in comparison to the Delta variant, Tixagevimab/Cilgavimab neutralizing titers were more markedly reduced against the BA.1 (344-fold) than against the BA.2 (9-fold) sublineages (46). In a study by Boschi et al. the virus-neutralizing activity of Tixagevimab/Cilgavimab was 233 times less active against the Omicron variant than against the Delta variant (44). This should be taken into account when developing new virus-neutralizing antibodies.

In our study, effective viral-neutralizing activity against the variants mentioned above was demonstrated by the negative results of PCR testing for SARS-CoV-2 RNA on Day 4 in the Tixagevimab/Cilgavimab groups being significantly more frequent than that in the Regdanvimab group (p < 0.017). PCR results in the Tixagevimab/Cilgavimab IV groups were 100% negative regardless of the dose used. It was also noted that the systemic inflammatory response, namely the levels of CRP and D-dimer, was significantly lower by Day 4 in the Tixagevimab/Cilgavimab 600 mg IV group than in other groups regardless of the concomitant anti-inflammatory therapy.

Upon IM administration of Tixagevimab/Cilgavimab, there was no increase in nAb titers immediately after administration due to the pharmacodynamics of mAbs, unlike with the IV administration. The nAb testing in this study was performed one hour after the drug administration and then on Day 4. The concentrations of mAbs in the Tixagevimab/Cilgavimab groups one hour after the administration were higher in the IV groups than those in the IM group, and became equal by Day 4, which is consistent with publication data (46–48). Upon the administration of equivalent doses of Tixagevimab/Cilgavimab IV and IM in the study by Bender Ignacio et al., the concentration of nAbs became equal by Day 3 (47). However, when administered IM, the absorption of the mAbs is influenced by many factors: body mass index, gender, and age, as well as excessive development of gluteal adipose tissue, especially when the drug is injected into adipose tissue, and not into muscles (46, 48). During SARS-CoV-2 infection, the time from the onset of symptoms to treatment may be important for mAbs and antivirals: some studies showed reduced efficacy in patients treated after 5 or 7 days (47). On average, the patients were hospitalized on Day 3 of the disease, however the nAbs concentration upon IM administration of Tixagevimab/Cilgavimab equaled the concentrations attained with the IV route of administration only by Day 8 from the COVID-19 onset. This may be indirectly related to the trend towards persisting positive PCR results on Day 4 after IM administration when compared with IV administration (13% versus 0%), as there is a delay in mAbs reaching the systemic circulation and exerting their neutralizing effect. Comparing the levels of nAbs to Omicron BA.5 sublineage in the blood sera of the patients with those of PCR testing of nasopharyngeal swabs on Days 0 and 4 of the study, we can conclude that IV administration of Tixagevimab/Cilgavimab in both studied doses can effectively neutralize the Omicron BA.5 sublineage of SARS-CoV-2 and reduce the number of PCR-positive patients as soon as Day 4 of the study.

## 5 Conclusion

Tixagevimab/Cilgavimab, in contrast to Regdanvimab, demonstrated higher nAbs titers to BA.1, BA.2, and BA.5 Omicron sublineages, as well as better laboratory efficacy and clinical results by Day 4 after the drug administration. The IV route of administration of Tixagevimab/Cilgavimab was associated with greater efficiency due to the faster effect. However, when compared with the nAbs titers to the Wuhan strain, a 22-fold decrease in virus-neutralizing activity was demonstrated, which suggests a possible loss of drug effectiveness due to the further mutations of SARS-CoV-2. Such rapid mutation makes it necessary to introduce new technologies into mAbs research and development.

## 6 Limitations

Limitations of this study include the lack of evaluation of virus neutralizing activity of serum after 4 days post-injection. There is also no evaluation of the effect of administered virus neutralizing antibodies on the development of long COVID-19.

## Data availability statement

The raw data supporting the conclusions of this article will be made available by the authors, without undue reservation.

## Ethics statement

The studies involving humans were approved by Local ethics committee City Clinical Hospital 52 (version 1.1 of 478 08.09.2022). The studies were conducted in accordance with the local legislation and institutional requirements. The participants provided their written informed consent to participate in this study.

## Author contributions

MSL: Writing – original draft, Writing – review & editing. DF: Writing – original draft, Writing – review & editing. AI: Writing – original draft. AKo: Writing – original draft. AS: Writing – original draft. SA: Writing – original draft. AC: Writing – original draft. ID: Writing – original draft, Writing – review & editing. TK: Writing – original draft. GA: Writing – original draft. AT: Writing – original draft. DS: Writing – original draft. AKa: Writing – review & editing. MAL: Writing – review & editing. DL: Writing – review & editing. AG: Writing – review & editing.
